# The effect of postmenopausal osteoporosis on subchondral bone pathology in a rat model of knee osteoarthritis

**DOI:** 10.1038/s41598-023-29802-7

**Published:** 2023-02-20

**Authors:** Hiroyuki Wada, Koji Aso, Masashi Izumi, Masahiko Ikeuchi

**Affiliations:** Department of Orthopaedic Surgery, Kochi Medical School, Kochi University, 185-1 Oko-cho Kohasu, Nankoku, 783-8505 Japan

**Keywords:** Experimental models of disease, Metabolic bone disease, Osteoarthritis

## Abstract

This study aimed to investigate the additional effect of ovariectomy-induced osteoporosis (OP) on the pathology of knee osteoarthritis (OA) in a rat meniscectomized model, particularly focusing on subchondral bone changes and pain behaviour. Rats were divided into four groups, sham, OP, OA, OP plus OA, and assessed for histology, osteoclast activity, subchondral bone microstructure, and pain-related behaviour. Rats with OP plus OA had significantly increased calcified cartilage and subchondral bone damage scores, increased densities of subchondral osteoclasts in the weight-bearing area, and more porous subchondral trabecular bone compared with rats with OA. Loss of tidemark integrity was observed most frequently in rats with OP plus OA. The density of subchondral osteoclasts correlated with the calcified cartilage and subchondral bone damage score in rats with OA (OA and OP plus OA). No significant differences in the receptor activator of nuclear factor-kappa B ligand (RANKL)/osteoprotegerin (OPG) expression ratio in subchondral bone and pain-related behavioural tests were observed between rats with OA and rats with OP plus OA. In rats with OA, coexisting OP potentially aggravated OA pathology mainly in calcified cartilage and subchondral trabecular bone by increasing subchondral osteoclast activity.

## Introduction

Knee osteoarthritis (OA) is a degenerative joint disease characterized by pathological features, including progressive cartilage damage, subchondral bone alterations, osteophyte development, and synovitis. In recent years, increasing evidence has suggested subchondral bone plays an important role in OA progression. Subchondral bone marrow lesions (BMLs) detected on magnetic resonance imaging (MRI) and elevated subchondral uptake in scintigraphy are associated with cartilage degeneration^1,2^. Furthermore, some studies have suggested that increased subchondral bone resorption is associated with early OA pathogenesis^3,4^. Subchondral bone metabolism may be altered depending on the OA stage and may affect the subchondral bone microstructure. Subchondral bone is divided into two parts: subchondral bone plate and subchondral trabecular bone. In patients with early OA, the subchondral bone plate becomes thinner and the subchondral trabecular bone becomes more porous. Conversely, in patients with late-stage OA, the subchondral bone plate becomes thicker, and the subchondral trabecular bone becomes denser^5^. The alteration of subchondral bone metabolism may exert a substantial effect on OA progression. Currently, osteoclasts, which play key roles in bone resorption, in subchondral bone have attracted increasing attention in OA pathogenesis. Several previous studies showed an increased number of subchondral osteoclasts in a rat OA model^6,7^ and human knees with OA^8^. Our previous study also revealed that osteoclast densities in subchondral bone were higher in symptomatic patients with knee OA^9^. Increased subchondral osteoclast activity might be one of the factors aggravating OA progression.

Osteoporosis (OP) is the most common disease in postmenopausal women^10^ and becomes more prevalent as people age^11^, similar to OA. Although an inverse relationship between OA and OP was proposed in previous large cohort studies^12–14^, recent studies have shown that positive^15^, negative^16^, and no^17^ association between OA and OP. The association is still being debated, but these two diseases share several risk factors, such as menopause and an advanced age^18^. As bone mineral density does not decreases in mild knee OA but decreases in advanced knee OA^16^, the coexistence of OA and OP is likely to become more common as the population ages. Subchondral osteoclast activity may promote OA development and/or progression, and thus the results from the few animal studies^19–21^ indicating that coexisting OP accelerates OA progression are reasonable. Meanwhile, the relationship between coexisting OP and OA progression remains controversial in human OA^12–14,22–24^.

The purpose of this study was to investigate the additional effect of OP induced by ovariectomy on knee OA pathology by particularly focusing on histological OA progression and pain behaviour in a rat model of medial meniscectomy-induced OA. We hypothesized that coexisting OP in the rat OA model would facilitate subchondral osteoclast activity and the subsequent progression of OA pathology.

## Results

### Knee OA histology

**Figure 1:**
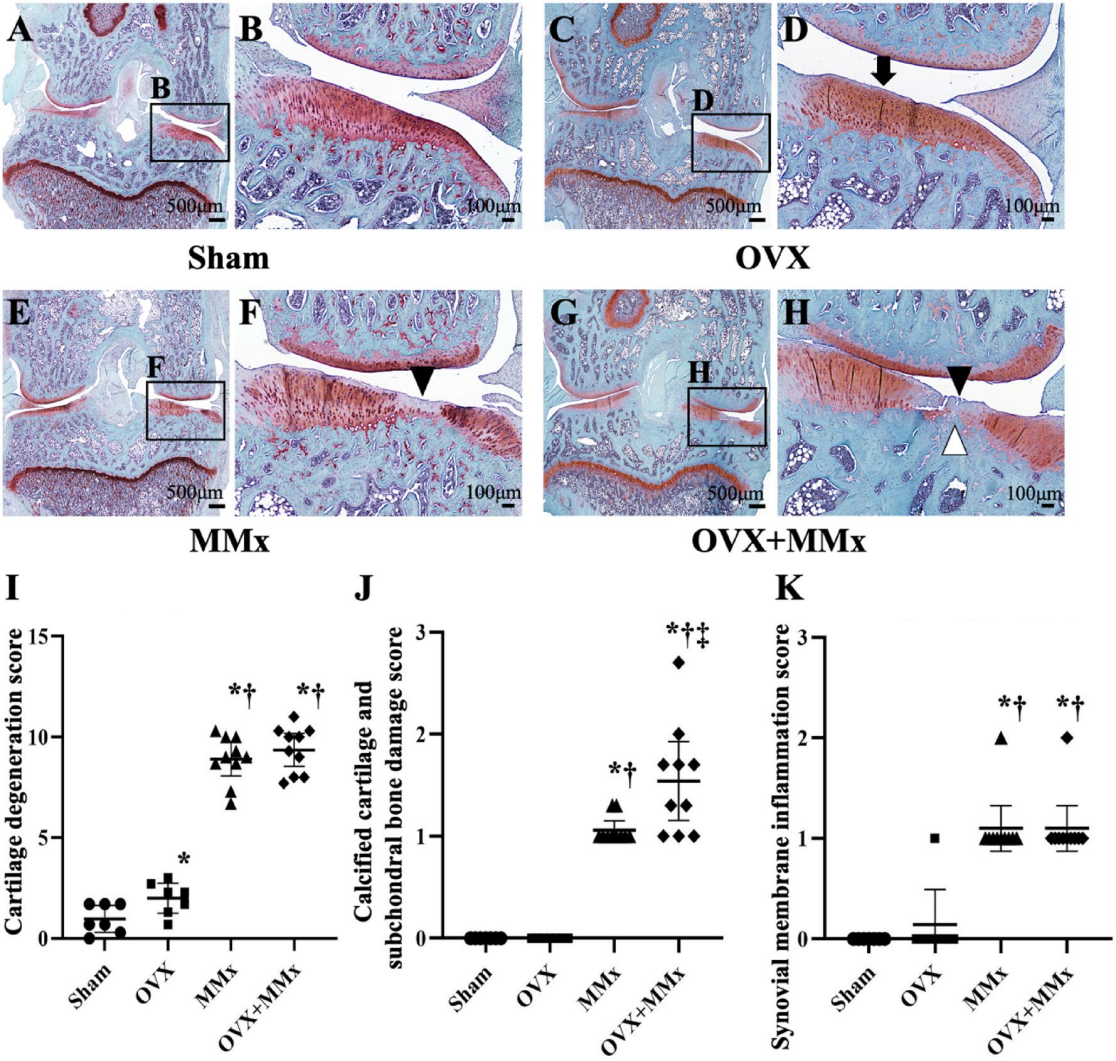
Histological analysis of cartilage degeneration. Histological findings of safranin-O/fast green staining in the Sham (**A**, **B**), OVX (**C**, **D**), MMx (**E**, **F**), and OVX + MMx (**G**, **H**) groups. Decreased safranin-O staining in the superficial layer of articular cartilage (arrow) was observed in the OVX group (D), and cartilage degeneration (black arrowhead) was observed in the MMx (F) and OVX + MMx (H) groups. Loss of tidemark integrity (white arrowhead) was observed in the OVX + MMx group (H). The right panels show higher magnification views of the boxed areas in the left panels. OARSI scores of each group (**I**, **J**, **K**). The calcified cartilage and subchondral bone damage scores of the OVX + MMx group were highest among the four groups (J). Lines represent the mean and 95% CIs. *: *p* < 0.05 compared with the Sham group; †: *p* < 0.05 compared with the OVX group; ‡: *p* < 0.05 compared with the MMx group.

The cartilage degeneration score, total Mankin score, and synovial membrane inflammation score of rats in the medial meniscectomy (MMx) (Fig. 1E,F) and bilateral ovariectomy plus medial meniscectomy (OVX + MMx) (Fig. 1G,H) groups were higher than those of rats in the Sham (Fig. 1A,B) and OVX (Fig. 1C,D) groups, while significant differences in these scores were not observed between the MMx and OVX + MMx groups (Table 1 and Fig. 1I,K). The cartilage degeneration score of rats in the OVX group was higher than that of rats in the Sham group, while a significant difference in the synovial membrane inflammation score was not observed between the groups (Table 1 and Fig. 1I,K). The calcified cartilage and subchondral bone damage score and tidemark integrity, which are the component scores of the Mankin score, in rats from the OVX + MMx group were highest among the four groups (Table 2 and Fig. 1J). The channel breaching tidemark was observed only in rats from the OVX + MMx group (Table 2). The subchondral bone plate thickness (BP.Th) in rats in the OVX group was lower than that in rats from the Sham group, while the value for rats from the MMx and OVX + MMx groups was higher than that for rats in the Sham and OVX groups (Table 2). No significant differences in BP.Th were observed between the MMx and OVX + MMx groups (Table 2).

**Table 1:** Mankin score of knee joints.

	Sham	OVX	MMx	OVX + MMx
Total Mankin score (range 0–14 points)	1.1 [0.6–1.7]	1.8 [1.4–2.3]	10 [9.6–10.4]*†	11.4 [10.4–12.3] *†
Structure (range 0–6 points)	0.2 [0–0.5]	0.3 [0–0.7]	3.9[3.5–4.2]*†	4.4 [3.9–5.0] *†
Cellularity (range 0–3 points)	0.2 [0–0.6]	0.7 [0.3–1] *	3 [3–3]*†	3 [3–3]*†
Proteoglycan degeneration (range 0–4 pints)	0.7 [0.4–1]	0.9 [0.6–1.1]	3.1 [2.9–3.3]*†	3.3 [3–3.7]*†
Tidemark integrity (range 0–1 point)	0 [0–0]	0 [0–0]	0 [0–0.1]	0.6 [0.3–0.9]*†‡

Values are presented as the means and 95% CIs. *: *p* < 0.05 compared with the Sham group; †: < 0.05 vs. OVX group; ‡: *p* < 0.05 compared with the MMx group.

**Table 2:** Density of channels breaching the tidemark and subchondral bone plate thickness.

	Sham	OVX	MMx	OVX + MMx
Density of channels breaching the tidemark, /mm	0 [0–0]	0 [0–0]	0 [0–0]	0.05 [0–0.12]
Subchondral bone plate thickness, μm	98.47 [94.59–102.35]	86.84 [80.3–93.38]*	112.5 [104.37–120.63]*†	115.17 [107.27–123.08]*†

Values are presented as the means and 95% CIs. *: p < 0.05 compared with the Sham group; †: *p* < 0.05 compared with the OVX group.

### Density of subchondral osteoclasts

**Figure 2:**
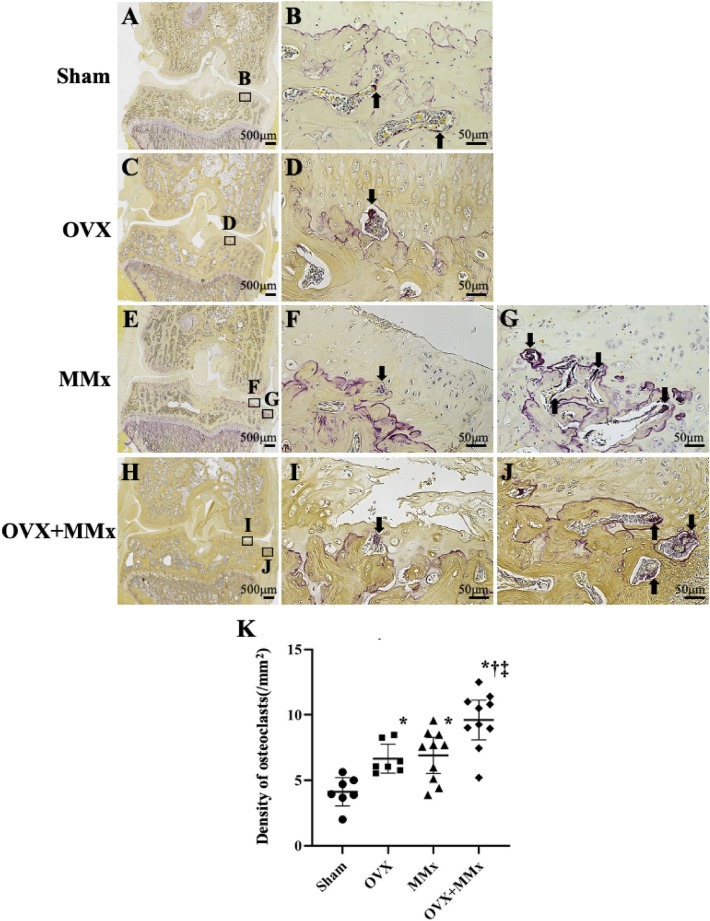
TRAP-positive osteoclasts in subchondral bone. Arrows show TRAP-positive osteoclasts. Osteoclasts resorbing calcified cartilage were observed in some sections (**D, F, I**) from all groups except for the Sham group (**B**). In addition, several osteoclasts were located around osteophytes (**G**, **J**). The middle and right panels show higher magnification views of the boxed areas in the left panels (**A, C, E, H**). The densities of subchondral osteoclasts in the OVX + MMx group were the highest among the four groups (**K**). Lines represent the mean and 95% CIs. *: *p* < 0.05 compared with the Sham group; †: *p* < 0.05 compared with the OVX group; ‡: *p* < 0.05 compared with the MMx group.

Subchondral osteoclasts resorbing calcified cartilage were observed among all rats except for those in the Sham group (Fig. 2A,B). Several subchondral osteoclasts were located around osteophytes (Fig. 2G,J). In rats from the OVX + MMx group (Fig. 2H,I,J), the highest density of subchondral osteoclasts was detected among the four groups (Fig. 2K). In rats from the OVX (Fig. 2C,D) and MMx groups (Fig. 2E,F,G), the densities of subchondral osteoclasts were higher than those in rats from the Sham group, while a significant difference in the densities of subchondral osteoclasts was not observed between the groups (Fig. 2K). The density of subchondral osteoclasts did not correlate with the cartilage degeneration score (r = 0.14 [−0.32, 0.55]) but correlated with the calcified cartilage and subchondral bone damage score (r = 0.60 [0.21, 0.82], *p* = 0.006) of rats in the MMx and OVX + MMx groups**.**

### sRANKL/OPG expression ratio in subchondral bone

**Figure 3:**
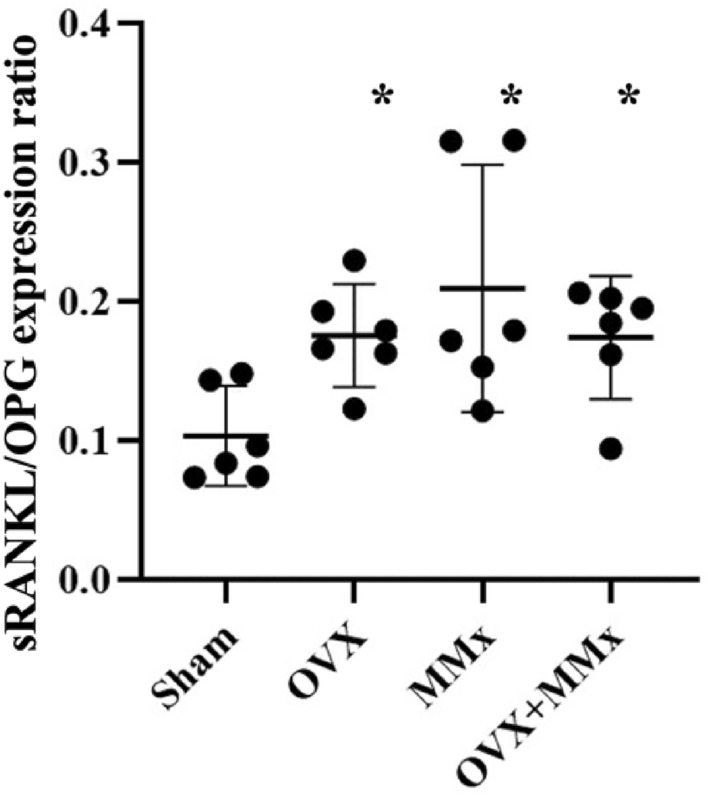
sRANKL/OPG expression ratio in subchondral bone. Lines represent the means and 95% CIs. *: *p* < 0.05 compared with the Sham group.

The soluble receptor activator of nuclear factor-kappa B ligand (sRANKL)/ osteoprotegerin (OPG) expression ratios in the OVX, MMX, and OVX + MMx groups were higher than those in rats from the Sham group. A significant difference in the sRANKL/OPG expression ratio was not observed between the rats in the OVX, MMX, and OVX + MMx groups (Fig. 3).

### Microcomputed tomography analyses of knee joints

**Figure 4:**
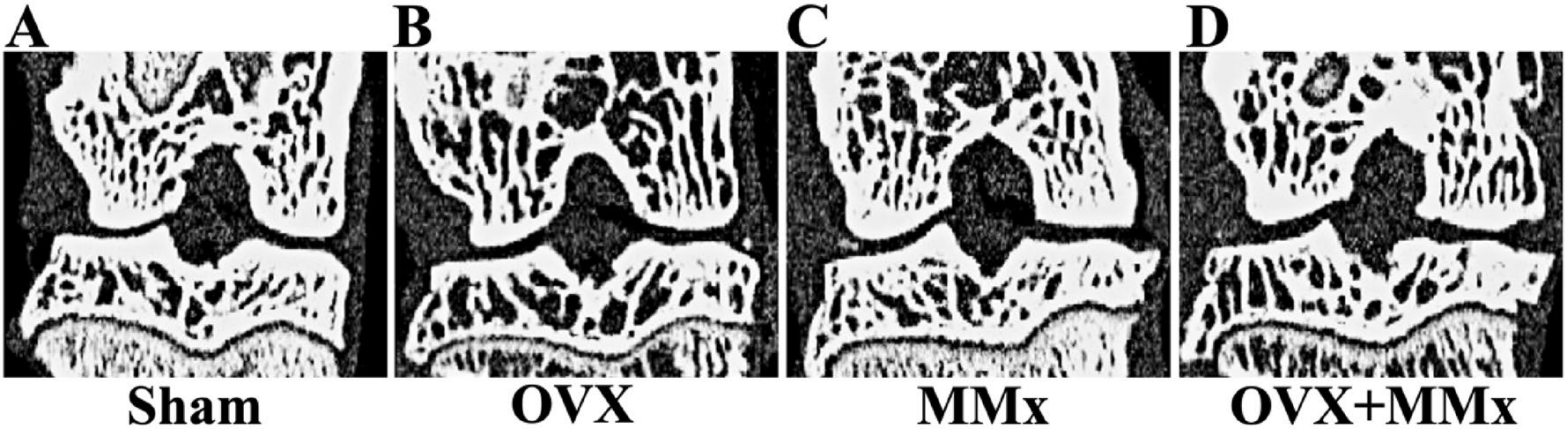
Microcomputed tomography images and bone mineral densities of knee joints (**A, B, C, D**). Osteoporotic changes were observed in the OVX rats (**B**). Subchondral sclerosis and osteophyte formation within the medial compartment were observed in the MMx and OVX + MMx rats (**C, D**).

The ovariectomized rats (Fig. 4B) exhibited osteoporotic changes compared with the Sham rats (Fig. 4A). In the bilateral knees of the OVX group and in the left knees of the OVX + MMx group, the bone volume fraction (BV/TV) and trabecular number (Tb.N) were lower and the trabecular spacing (Tb.Sp) was higher than in the bilateral knees of the Sham and MMx groups (Table 3). Osteophyte formation within the medial compartment was observed in the meniscectomized rats (Fig. 4C,D) and the subchondral trabeculae bone in the right knees of the MMx group was denser. In the right knees of the MMx group, the BV/TV and trabecular thickness (Tb.Th) were higher than those in the right knees of the Sham group (Table 3). The subchondral trabecular bone in the right knees of the OVX + MMx group was denser than that of the OVX group and more porous than that of the MMX group. The right knees of the OVX + MMx group exhibited a higher BV/TV and lower Tb.Sp than those in the OVX group. On the other hand, the BV/TV was lower and the Tb.Sp was higher than that in the MMx group (Table 3). The paired t test did not reveal significant differences between the subchondral trabecular bone microstructural parameters in the bilateral knees of the Sham and OVX groups. In the right knees of the MMx and OVX + MMx groups, the BV/TV and Tb.Th were higher and the Tb.Sp was lower than that of the left knees.

**Table 3:** Microcomputed tomography evaluation of subchondral bone.

	Sham	OVX	MMx	OVX + MMx
Right knee (Intervention)				
BV/TV, %	61.66 [60.53–62.79]	58.08 [56.82–59.34]*§	65.17 [64.17–66.17]*†§||	60.97 [59.56–62.37] ||
Tb.Th, μm	104.25 [100.49–108.01]	103.99 [98.71–109.26]	117.02 [112.68–121.36]*||	110.1 [104.91–115.29] ||
Tb.Sp, μm	156.31 [147.12–165.51]	190.24 [172.19–208.28]*§	143.89 [134.62–153.16] †§||	168.79 [157.6–179.98] ||
Tb.N, mm^-1^	3.84 [3.71–3.98]	3.42 [3.18–3.65]*‡	3.85 [3.68–4.01]	3.6 [3.46–3.73]
Left knee (Control)				
BV/TV, %	61.49 [60.2–62.77]	59.27 [56.77–59.78]*‡	61.31 [60.47–62.16]	57.62 [56.32–58.91]*‡
Tb.Th, μm	105.64 [100.77–110.5]	102.69 [97.62–107.76]	106.11 [101.47–110.76]	102.84 [97.76–107.92]
Tb.Sp, μm	158.9 [156.22–161.58]	187.58 [170.21–204.95]*‡	156.11 [151–161.21]	183.2 [174.04–192.35]*‡
Tb.N, mm^-1^	3.8 [3.75–3.85]	3.46 [3.21–3.71]‡	3.82 [3.72–3.92]	3.5 [3.38–3.62]*‡

Values are presented as the means and 95% CIs. *: *p* < 0.05 compared with the Sham group; †: *p* < 0.05 compared with the OVX group; ‡: *p* < 0.05 compared with the MMx group; §: *p* < 0.05 compared with the OVX + MMx group; ||: *p* < 0.05 compared with the left knee.

### Pain-related behaviour tests

**Figure 5:**
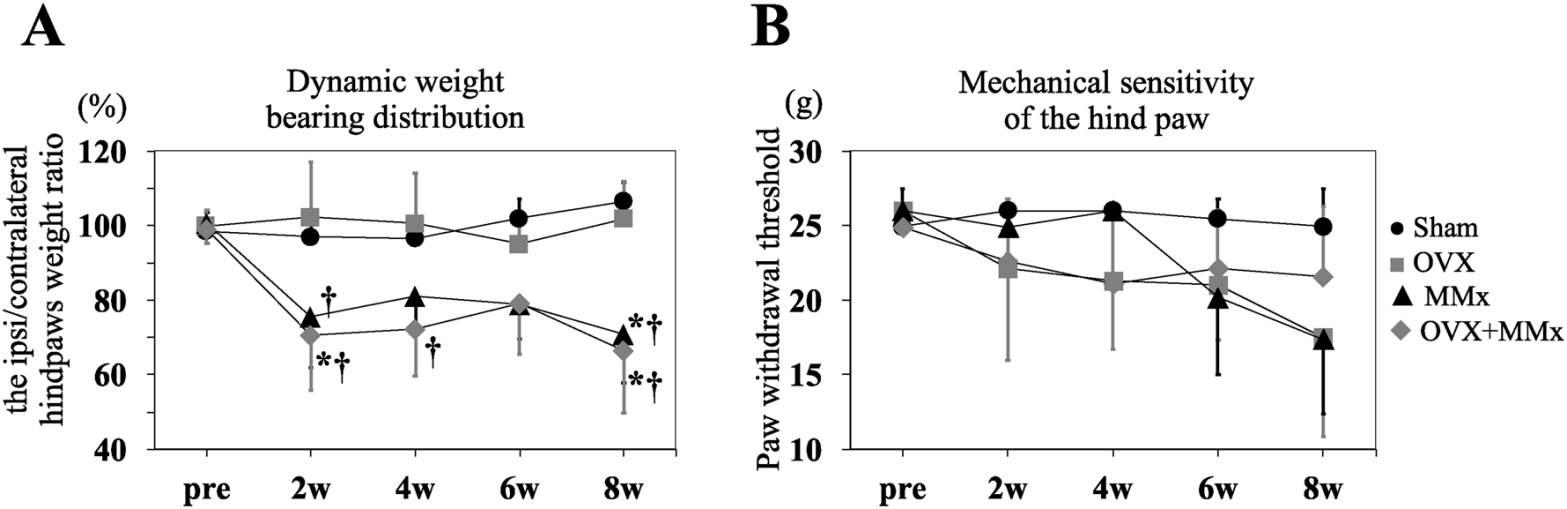
Pain-related behavioural tests. The pain-related behaviours were assessed using the dynamic weight bearing test (**A**) and von Frey test (**B**). Lines represent the mean and 95% CIs. *: *p* < 0.05 compared with the Sham group; †: *p* < 0.05 compared with the OVX group.

The ipsi/contralateral hind paw weight ratios of the MMx and OVX + MMX groups were decreased at 2 weeks and further deteriorated at 8 weeks compared with those of the Sham and OVX groups (Fig. 5A). The mechanical sensitivity of the hind paw in rats from the OVX and OVX + MMx groups showed a downwards trajectory from 2 weeks, while the mechanical sensitivity of the hind paw in rats from the MMx group showed a similar tendency from 6 weeks (Fig. 5B). However, no significant differences were observed among any of the groups at any time-point.

## Discussion

Our results showed that OA rats with OP had higher calcified cartilage and subchondral bone damage scores, increased densities of subchondral osteoclasts, and more porous subchondral trabecular bone than OA rats. Loss of tidemark integrity was observed most frequently in the OA rats with OP. Cartilage degeneration scores, total Mankin scores, synovial membrane inflammation scores, BP.Th, and the sRANKL/OPG expression ratio in subchondral bone were not different between the OA rats with and without OP. Based on these results, coexisting OP in rats with OA aggravates OA pathology mainly in calcified cartilage and subchondral trabecular bone.

The effect of OA on subchondral bone microstructure is opposite to that of OP. In the present study, OA rats had thicker subchondral bone plates and denser subchondral trabecular bone, while postmenopausal OP rats displayed thinner subchondral bone plates and more porous subchondral trabecular bone. These results are consistent with previous studies of rat models^25,26^ and humans^27,28^. In OA rats with OP, which exerted two contrasting effects on the subchondral bone microstructure, subchondral trabecular bone became more porous than in OA rats. However, a significant difference in BP.Th was not observed between OA rats with and without OP. Coexisting OP in OA rats primarily affected the subchondral trabecular bone but not the subchondral bone plate, as previously reported in OA mice with OP^29^. Subchondral trabecular bone would be more important in terms of subchondral bone stiffness because a parametric finite element modelling study^30^ suggested that the stiffness of the human proximal tibia was affected primarily by epiphyseal subchondral trabecular bone rather than the subchondral bone plate. Coexisting OP in subjects with OA aggravates OA pathology due to the deteriorated biomechanical properties of subchondral trabecular bone, consistent with a previous study^31^.

To the best of our knowledge, this study is the first to evaluate the sRANKL/OPG expression ratio in subchondral bone. Our results showed inconsistent results for the sRANKL/OPG expression ratio in subchondral bone and the density of subchondral osteoclasts in the weight-bearing area: the sRANKL/OPG expression ratio in OA rats with OP was similar to that in OA rats, while the density of subchondral osteoclasts in OA rats with OP was higher than that in OA rats. The reason why the additional effect of OP on osteoclast activity was not apparent in the sRANKL/OPG expression ratio in subchondral bone might be that measured area included a nonweight-bearing area. Previous studies have shown that mechanosensing ion channels are expressed at high levels during osteoclast differentiation^32^, and high loading stress increases the osteoclast number^33^. Because the subchondral bone of OA rats with OP was more porous and more vulnerable to mechanical load than that of OA rats, loading stress exerted a stronger effect on osteoclast activity in the weight-bearing subchondral bone of OA rats with OP. The additional effect of OP on subchondral osteoclast activity in OA rats might occur mainly in the weight-bearing area. Our results also showed that the densities of subchondral osteoclasts correlated with calcified cartilage and subchondral bone damage scores but not with cartilage degeneration scores. In addition, subchondral osteoclasts resorbing calcified cartilage were observed among rats except the Sham group, and channel breaching tidemarks were observed only in the OA rats with OP. A previous in vitro study indicated that osteoclasts could resorb noncalcified and calcified cartilage^34^. Furthermore, resorption pits reaching from subchondral bone into articular cartilage have been observed in OA patients, and osteoclast-like cells attaching to the inner surface of the pit seemed to absorb calcified cartilage and articular cartilage^35^. Based on these results, coexisting postmenopausal OP in rats with OA might promote the resorption of calcified cartilage due to increased numbers of subchondral osteoclasts in the weight-bearing area and aggravate OA pathology.

In our study, coexisting postmenopausal OP in OA rats accelerated the progression of OA pathology. Previous reports also showed that subchondral bone loss induced by OVX accelerated the progression of OA pathology in rodents^21,29,36,37^ and rabbits^19^. However, rats with postmenopausal OP alone only echibited a reduction in safranin O staining in the superficial layer of articular cartilage. In postmenopausal OP rats, the increase in osteoclast activity was the same as that in OA rats, and subchondral osteoclasts resorbing calcified cartilage were observed as in OA rats. However, the extent of OA progression was similar to that in Sham rats. Based on these results, the state of increased subchondral osteoclast activity followed by subchondral bone loss induced by OVX was not a main causal factor but an accelerator of the progression of OA pathology. Additional factors, such as increased loading stress in the articular cartilage induced by MMx in this study, might be needed to promote OA progression in postmenopausal OP rats.

The underlying mechanism affecting neural processing of pain might differ between OA and postmenopausal OP. Subchondral osteoclasts induced sensory nerve innervation in subchondral bone by secreting Netrin-1 in a rat OA model^38^, and osteoclasts are the source of nerve growth factor, which sensitizes primary afferents^9^. In postmenopausal OP, oestrogen deficiency may affect pain signal transduction. Oestrogen receptors are distributed widely in the central nervous system and dorsal root ganglion (DRG)^39,40^. Postmenopausal OP rats show mechanical hypersensitivity of the hind paw, which may be reversed by oestrogen replacement therapy^41^. In this study, a downwards trajectory of the paw withdrawal threshold was observed at the early phase in postmenopausal OP rats, while a similar trend was detected at the later phase in OA rats. The sudden loss of serum oestrogens induced by OVX might affect pain processing and cause early mechanical hypersensitivity in the hind paw. Meanwhile, the development of mechanical hypersensitivity in OA rats might be associated with OA progression. Mechanical hypersensitivity might be established only in the late phase of OA^42^. In weight bearing pain, the effect of OA induced by MMx might be much stronger than that of oestrogen deficiency, and thus the additional effect of OVX could not be detected.

Our study suggested the hyperactivity of subchondral osteoclasts in the weight-bearing area was associated with OA progression and revealed the possible effect of antiresorptive drugs, such as bisphosphonate, denosumab, oestrogens, and selective oestrogen receptor modulators (SERMs) on preventing OA progression. According to some reports, both bisphosphonate and oestrogen use protect against arthroplasty^43,44^ and both drugs are associated with significantly less subchondral bone attrition and BMLs in humans^45^. SERMs also suppress cartilage degradation in both rodents and humans^46^. However, at present, evidence that antiresorptive drugs prevent OA progression is insufficient. A recent meta-analysis of randomized controlled trials assessing knee OA showed that bisphosphonates neither provided symptomatic relief nor suppressed radiographic progression^47^. One potential explanation is that OA is a heterogeneous syndrome with different clinical phenotypes. The subchondral osteoclast activity in different OA stages varies from patient to patient. A previous study showed that bisphosphonate therapy is effective in nonoverweight postmenopausal women with early-stage OA^48^. We postulate that antiresorptive drugs are beneficial for certain subsets of OA patients with hyperactivity of subchondral osteoclasts. Further studies are needed to identify the proper drug and time for antiresorptive drug administration to prevent OA progression.

This study has several potential limitations. First, this study was observational, and further interventional studies using antiresorptive drugs are needed to clarify whether hyperactivity of subchondral osteoclasts is associated with OA pathology. Second, OA histology was assessed in our study only at a single time point after the operation. The follow-up periods were not very long, but 8 weeks would be sufficient because bone mineral density started to decrease at 2 weeks^49^ and further decreased at 4 and 8 weeks in the ovariectomized rat OP model^50^. We confirmed that severe cartilage degeneration occurred within 6 weeks with full thickness involvement in the tidemark in a medial meniscectomy-induced rat OA model^51^. Studies performing assessments at multiple time points are needed in the future. Third, the difference in the timing of OVX might affect the progression of OA pathology. However, even the OA rabbit knees with OP^19,52^, in which OVX was performed before meniscectomy, showed similar results to our findings: coexisting OP in the OA model aggravated OA pathology. The difference in the timing of OVX might have little effect on OA pathology.

In conclusion, coexisting OP in OA rats potentially aggravated OA pathology mainly in calcified cartilage and subchondral bone of the weight-bearing area due to increased osteoclast activity. Our results suggest that antiresorptive therapy has promising potential to prevent OA progression in certain subsets of OA patients with OP.

## Methods

### Animals

Female Sprague–Dawley rats (12 weeks old, weight 210–250 g; n = 60) were used in this study in accordance with the International Association for the Study of Pain guidelines. The animals were maintained at the Institute of Laboratory Animals, Kochi Medical University. They were housed in a temperature-controlled room at 22 ± 3 °C on a 14-h/10-h light/dark cycle and were provided ad libitum access to food and water. No more than three rats were housed in each cage to ensure that they were sufficiently active and to avoid biting. The rats were divided into four groups: sham surgery (Sham group; n = 13), bilateral ovariectomy (OVX) (OVX group; n = 13), medial meniscectomy (MMx) of the right knee (MMx group; n = 17), and bilateral OVX plus MMx of the right knee (OVX + MMx group; n = 17) groups. Two rats (MMx: 1, OVX + MMx: 1) were excluded from the series because of postsurgical joint infection. Six rats from each group were used for enzyme-linked immunosorbent assays (ELISAs). The other rats from each group were used for histology, microcomputed tomography, and pain-related behaviour tests. All experiments were approved by the Animal Care and Use Committee of Kochi University (O-00010). The study was performed in compliance with ARRIVE guidelines.

### Ovariectomy and medial meniscectomy

Prior to the experiments, the rats were allowed to adapt to the laboratory environment for 1 week. The animals were anaesthetized with 2–3% isoflurane before undergoing surgery. All rats received three incisions on the same day: bilateral dorsolateral incisions and one right knee incision. Bilateral OVX was performed using a dorsolateral approach^53^ to induce OP, and MMx was performed via an incision on the medial aspect of the right knee joint capsule along with transection of the medial collateral ligament (MCL)^54^ to induce OA. The MCL of the right knee was exposed but was not transected in the OVX group, and bilateral ovaries were exposed but were not removed in the MMx group. The bilateral ovaries and MCL of the right knee were exposed in the Sham group. After the surgical procedure, the muscle and skin were sutured.

### Histology

The animals were sacrificed by CO_2_ overdose at 8 weeks postsurgery. Bilateral tibiofemoral joints were collected and placed in neutral buffered formalin (4% formaldehyde), decalcified in 10% ethylenediaminetetraacetic acid (EDTA) for 8 weeks and embedded in paraffin wax. For pathological measurements, 4 μm-thick paraffin frontal sections were cut throughout the middle third of the medial tibiofemoral joint. The sections were stained with haematoxylin–eosin (H&E), safranin-O, fast green, and tartrate-resistant acid phosphatase (TRAP). TRAP staining was performed using a commercially available kit (Sigma–Aldrich 387A).

Sections were viewed with a BZ-X800 all-in-one fluorescence microscope (KEYENCE, Japan). The cartilage degeneration score, calcified cartilage and subchondral bone damage score, and synovial membrane inflammation score of the right medial tibial plateau were assessed according to the Osteoarthritis Research Society International (OARSI) grading system criteria^55^ and the Mankin scoring system^56^. In the OARSI criteria, the calcified cartilage and subchondral bone damage score and synovial membrane inflammation score were calculated using a numerical scale ranging from 0 (best) to 5 (worst) points. The most severe lesion of the right medial tibial plateau on each frontal section was scored. For the cartilage degeneration score, the medial tibial plateau on each frontal section was divided into three zones of equal width, and cartilage degeneration in each zone was scored from 0 (best) to 5 (worst) points. The total cartilage degeneration score was calculated by adding the values obtained for each zone. The maximum cartilage degeneration score was 15. Cartilage damage in the right medial tibial plateau was also quantified with the Mankin scoring system. Using this scoring system, the maximum score obtainable was 15. The BP.Th of the right medial tibial plateau was also assessed using the same method as described in a previous report^57^. The BP.Th area was defined as the area between the osteochondral junction and the bone marrow cavity. The mean BP.Th was calculated by dividing the area of the subchondral bone plate by the length of the bottom. The numbers of TRAP-positive osteoclasts were quantified on the right medial tibial epiphyses. TRAP-positive osteoclasts were quantified under 40 × magnification from one end of the growth plate to the other end of the medial tibial plateau using the following criteria: 1) displayed a purplish to dark red cytosol, 2) ≥ 3 nuclei/osteoclast, and 3) located within the subchondral bone area, comprising the area between the cartilage/bone junction and the growth plate. Lightly stained TRAP-positive cells without nuclei identified or located distal to the subchondral bone were not counted. The subchondral bone area of the medial tibial plateau was manually outlined, and the area was measured with BZ-X800 Analyser software (KEYENCE, Japan). The density of osteoclasts in the epiphysis of the medial tibial plateau was calculated. Histological scoring, except for the synovial membrane inflammation score, was performed on the three most severely affected consecutive sections. The values for each parameter were then averaged across the three scored sections per knee joint. The synovial membrane inflammation score was determined for one section.

### ELISA

The medial compartments of the right proximal tibia and distal femur were cut 5 mm from the joint line, weighed, and immediately stored at −80 °C. These samples were cut into small pieces with a bone cutter and crushed with extraction buffer, containing ice-cold phosphate-buffered saline (PBS), pH 7.4, protease inhibitors (cOmplete Tablets, Mini EASYpack, Roche) and 0.1% Triton X-100 using a Precellys Evolution homogenizer (Bertin Instruments, Rockville, MD, USA). The amount of added extraction buffer was determined by the weight of each sample to adjust the difference in sample volume. Soluble receptor activator of nuclear factor-kappa B ligand (sRANKL) and osteoprotegerin (OPG) expression levels were quantified and analysed using a sandwich ELISA kit for sRANKL and OPG (Rat sRANKL ELISA kit; Immundiagnostik AG, Rat Osteoprotegerin ELISA kit; Immundiagnostik AG, Bensheim, Germany) according to the manufacturer's protocols. The sRANKL/OPG expression ratio, which suggests increased osteoclast activity^58^, was calculated.

### Microcomputed tomography

The proximal tibial epiphyses of the bilateral knees were scanned using a micro-CT system (Latheta LCT-200, Hitachi-Aloka Medical, Tokyo, Japan) with a pixel size of 24 × 24 μm and slice thickness of 75 μm, which was operated at 50 kV and 0.5 mA. All knees were placed in the 24-mm specimen holder to prevent movement. Thresholds for segmenting bone/soft tissue and trabecular/cortical bone were set to 500 and 1200 mg/cm^3^ respectively using Latheta software version 3.3. Subchondral bone in the medial tibial epiphysis was chosen as the volume of interest (VOI; mm^3^) (see Supplementary Table S1 online). Cortical bone was excluded from the VOI. Then, the following subchondral trabecular bone microstructural parameters were calculated: BV/TV (%), Tb.Th (μm), Tb.Sp (μm) and Tb.N (/mm^-1^). BV/TV was measured using Latheta software version 3.3. Tb.Th and Tb.Sp were measured using Fiji software^59^ and the BoneJ plugin^60^, which are Java-based image processing programs developed at the National Institutes of Health and the Laboratory for Optical and Computational Instrumentation. The results of Tb.Sp and Tb.Th were used to calculate Tb.N with the following formula 1/(Tb.Th + Tb.Sp)^61^.

### Pain-related behavioural tests

Two separate measures of pain-related behaviour were assessed before surgery and 2, 4, 6, and 8 weeks after surgery: the change in dynamic weight-bearing distribution^62^ and mechanical sensitivity of the hind paw^63^. The distribution of weight bearing on the hind paw was measured using a dynamic weight bearing test 2.0 (DWB2) (BioSeb, Vitrolles, France). During testing, the animals moved freely for 5 min in the test box, where the 22 cm × 22 cm floor consisted of a mat with force sensors indirectly registering the weight of each paw. The rats were simultaneously filmed from above with a high-definition camera to facilitate subsequent validation of paw placement. The filmed paw prints and the corresponding weight exerted by each limb on the sensor pad were automatically processed with DWB software version 2.0.63 (BioSeb). The software partitioned the five-minute video into analysable and nonanalysable segments. A minimum of 1.5 min of analysable time was required to meet the analysis thresholds. After the manual attribution of each pressure point to the corresponding paw, the ipsilateral/contralateral hind paw weight ratio was extracted from the acquired data. The mechanical sensitivity of the right hind paw was examined using von Frey filaments. Rats were placed inside a plexiglass cage placed on an elevated mesh steel platform. von Frey filaments with varying bending forces (0.4, 0.6, 1, 1.4, 2, 4, 6, 8, 10, 15, and 26 g) were applied to the plantar surface of the right hind paw in ascending order of bending force. Each filament was applied three times for approximately 2–3 s periods or until a withdrawal response was evoked. After a response, the hind paw was retested with the filaments in descending order until no response occurred, at which point the filaments were again applied in ascending order until the response was once again evoked. The final bending force that induce leg withdrawal was recorded three times. The average value was recorded as the mechanical threshold of the paw.

### Statistical analysis

All data are presented as the mean values and 95% confidence intervals (CIs). Statistical analyses were performed with JMP, version 10 (SAS Ins. Cary, NC), IBM SPSS version 26.0 software and IBM SPSS Bootstrapping (IBM Corp. Armonk, NY, USA). The variables of histological results, ELISA results, and subchondral bone microstructural parameters were analysed using one-way ANOVA followed by Tukey’s post hoc test or Games-Howell post hoc test. A paired t test was used to determine the differences between the subchondral bone microstructural parameters of the right knees and left knees in each group. The sRANKL/OPG expression ratio was compared using one-way ANOVA with the bootstrapping method. Pearson’s moment correlation coefficient (r) was calculated to examine the relationship between the OARSI score and the density of subchondral osteoclasts. Pain behaviours were analysed using two-way ANOVA followed by Tukey’s post hoc test. P < 0.05 indicated statistical significance.

## Supplementary Information

Supplementary Information.

## Data Availability

The data that support the findings of this study are available from the corresponding author, K.A., upon reasonable request.
