# Supplementary material for: The effect of postmenopausal osteoporosis on subchondral bone pathology in a rat model of knee osteoarthritis
Source: Sci Rep. 2023 Feb 20;13:2926. doi: 10.1038/s41598-023-29802-7 (PMC9941090; doi:10.1038/s41598-023-29802-7)
Supplement: Supplementary file 1 — Supplementary Information. [file 41598_2023_29802_MOESM1_ESM.docx]

|  | **Sham** | **OVX** | **MMx** | **OVX+MMx** |
| --- | --- | --- | --- | --- |
| Right knee, mm^3^ | 13.22 [12.43-14.00] | 12.93 [11.76-14.10] | 12.19 [11.49-12.88] | 12.69 [12.00-13.38] |
| Left knee, mm^3^ | 13.24 [12.44-14.05] | 13.21 [12.38-14.05] | 12.99 [12.36-13.61] | 13.35 [12.72-13.97] |

**Supplementary Table S1.** The sizes of the VOIs in the subchondral trabecular bone

Values are presented as the means and 95% CIs. No significant differences were observed among any of the groups.
